# Temporal observation of endoscopic and histological findings of gastritis after administration of an immune checkpoint inhibitor: a case report

**DOI:** 10.1007/s12328-021-01582-5

**Published:** 2022-01-05

**Authors:** Keita Saito, Daiki Ozono, Hironobu Nagumo, Masayo Yoshimura, Yoko Masuzawa

**Affiliations:** 1 Department of Gastroenterology, Kameda General Hospital, 929, Higashi-cho, Kamogawa, Chiba 296-8602 Japan; 2 Department of Anatomic Pathology, Kameda General Hospital, Chiba, Japan

**Keywords:** Immune checkpoint inhibitor, Pembrolizumab, Immune-related adverse events

## Abstract

A 71-year-old Japanese man was treated with 200 mg of pembrolizumab for lung adenocarcinoma with multiple bone metastases at the Department of Respiratory Medicine of Kameda General Hospital. After 19 treatment courses, he complained of epigastric pain before meals. Upper gastrointestinal endoscopy showed multiple erosions in the gastric antrum, and antacids were administered at follow-up. After 27 treatment courses, the patient underwent another endoscopy because of anorexia. The erosions were enlarged and had increased from the gastric antrum to the greater curvature of the body. Histological biopsy showed lymphocytic infiltration with a predominance of CD8-positive T cells. The patient had previously been treated for *Helicobacter pylori* infection, and we suspected drug-induced gastritis due to the administration of immune checkpoint inhibitors in the course of the disease. Pembrolizumab was discontinued, and the patient’s symptoms gradually improved. Endoscopic examinations were performed 2, 5, and 9 months after discontinuation of pembrolizumab, and improvement in mucosal findings and decreased lymphocyte infiltration were confirmed each time. The patient has remained without any relapse of symptoms for more than 1 year after discontinuing treatment.

## Introduction

Immune checkpoint inhibitors (ICIs) are widely used in the treatment of various cancers, including gastric and esophageal cancers. ICIs are monoclonal antibodies that target negative immunomodulatory molecules, such as cytotoxic T-lymphocyte antigen 4 (CTLA-4) receptors, the programmed cell death 1 (PD-1) receptor, and the PD-1 ligand (PD-L1). Pembrolizumab is a PD-1 antibody that is an effective treatment for un-resectable advanced lung cancer [1]. The adverse effects of ICIs are known as immune-related adverse events (irAEs), and many reports on irAEs, such as colitis and hepatitis, have already been published. However, there have been few reports on gastritis, and the characteristics of endoscopic findings and changes over time have not been clearly elucidated. Herein, we present a case of gastritis that developed after the administration of pembrolizumab. We report the temporal changes in symptoms, affected sites, and endoscopic findings of gastritis after ICI administration through comparison with previous case reports.

## Case report

A 71-year-old Japanese man with un-resectable advanced lung adenocarcinoma was started on a once-every-three-week regimen of 200 mg pembrolizumab, a PD-1 inhibitor, at the Department of Pulmonary Medicine in our hospital in 2019. At that time, the patient was 167 cm tall and weighed 63 kg. He had a history of pulmonary tuberculosis, chronic obstructive pulmonary disease, and hypertrophic cardiomyopathy. His only oral medication was bisoprolol fumarate for cardiac disease. He had no food or drug allergies and consumed approximately 20 g of alcohol and 15 cigarettes per day. After 19 cycles of treatment, the patient developed epigastric pain and underwent upper gastrointestinal endoscopy, which revealed multiple erosions in the gastric antrum. Antacid treatment was started, and the patient was followed up for approximately 20 weeks; however, after 27 courses of treatment, he became anorexic. Blood tests showed no elevation of white blood cells or a decrease in hemoglobin in the peripheral blood, and both renal and liver functions were normal. When endoscopy was performed again, the erosions observed in the previous examination had spread extensively from the gastric antrum to the gastric body, and the number of erosions had increased.

**Fig. 1:**
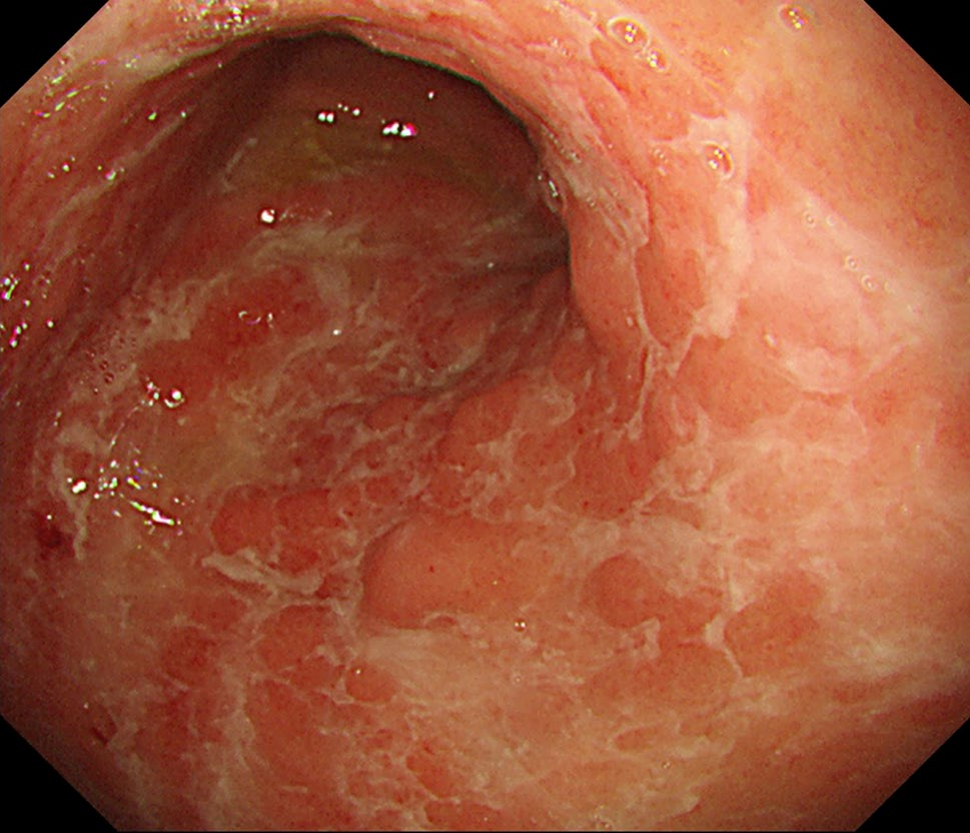
Gastrointestinal endoscopic image after 27 courses of pembrolizumab administration. The patient had only minor symptoms of anorexia, but diffuse erythema, erosion, and white exudate from the gastric antrum to the body were observed

**Fig. 2:**
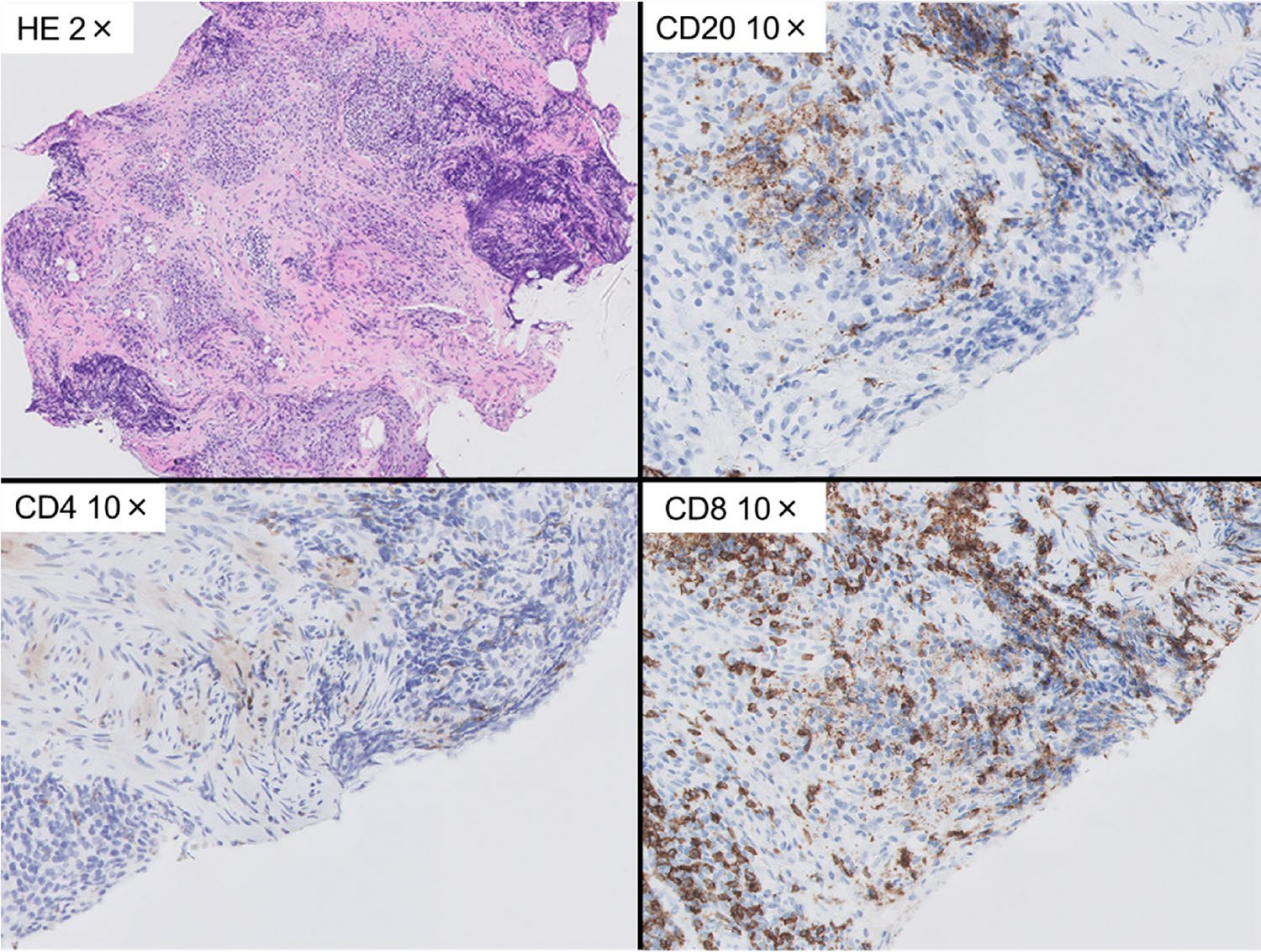
Histological images of the gastric mucosa after 27 courses of pembrolizumab administration. Many plasma cells and lymphocytic infiltrates were observed. Immuno-histochemical staining showed that CD20 and CD4 were also positive, but the number of CD8-positive T cells was predominantly increased (287 per field of high magnification)

Endoscopic examination of the stomach revealed extensive erosions and erythema with solid white exudate from the gastric antrum to the greater curvature of the gastric body (Fig. 1). No abnormal findings were noted in the esophagus or duodenum. Tissue biopsy of the gastric erosion showed inflammatory cell infiltration into the interstitium, mainly lymphocytes and plasma cells, as well as thickening of the muscularis mucosae and granulation tissue (Fig. 2). Immunohistochemistry analysis revealed increased numbers of both CD4-positive T cells and CD8-positive T cells, but the increase in CD8-positive T cells was particularly dominant. Furthermore, there were no other findings that would raise suspicion of malignant diseases such as cancer or lymphoma.

**Fig. 3:**
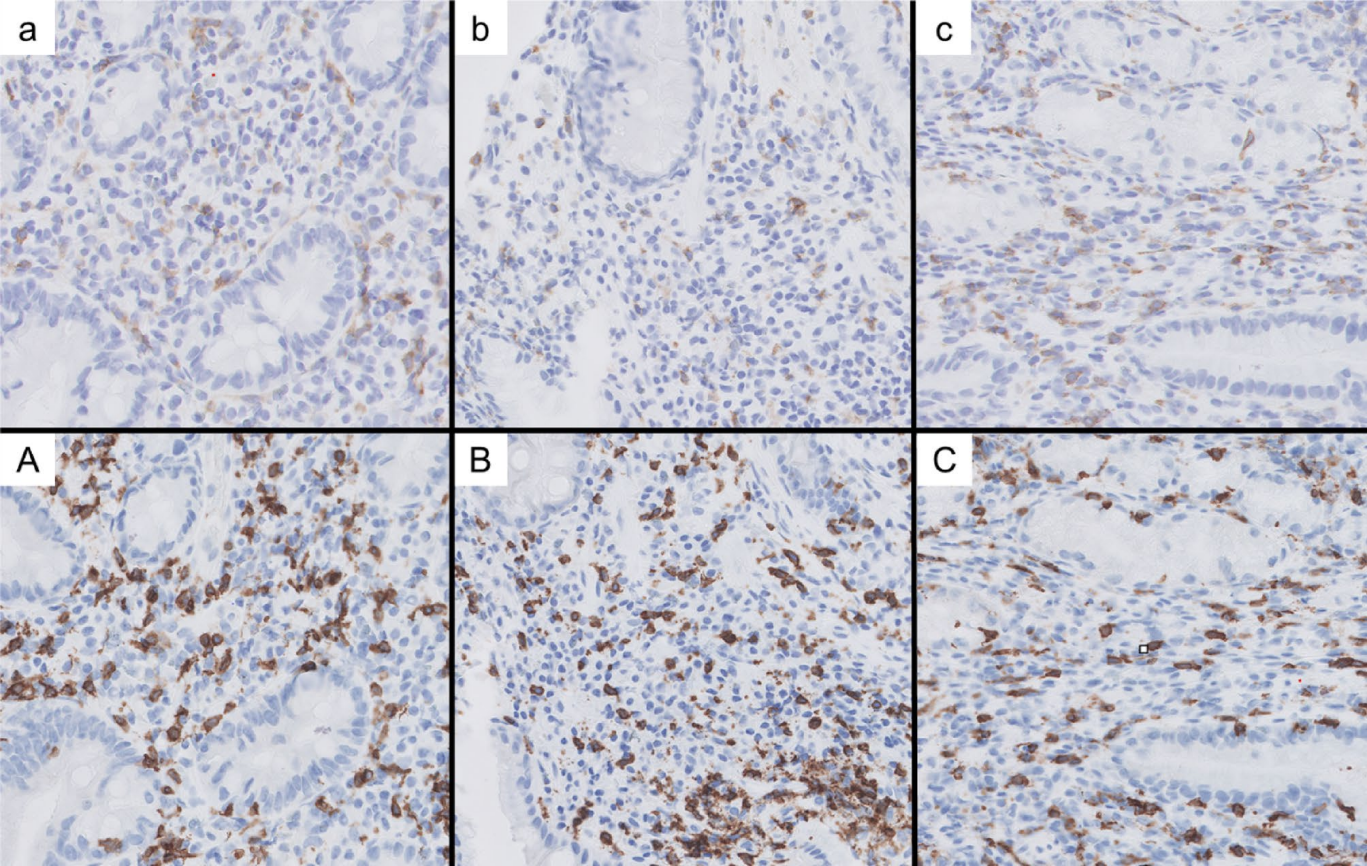
Histological images of immuno-histochemical staining of gastric mucosa 2 months (**a** CD4; **A** CD8), 5 months (**b** CD4; **B** CD8), and 9 months (**c** CD4; **C** CD8) after discontinuation of pembrolizumab, respectively (objective magnification × 10). The number of CD4-positive T cells did not change significantly, but the number of CD8-positive T cells decreased over time to 217, 201, and 104 per field of high magnification at 2, 5, and 9 months, respectively

**Fig. 4:**
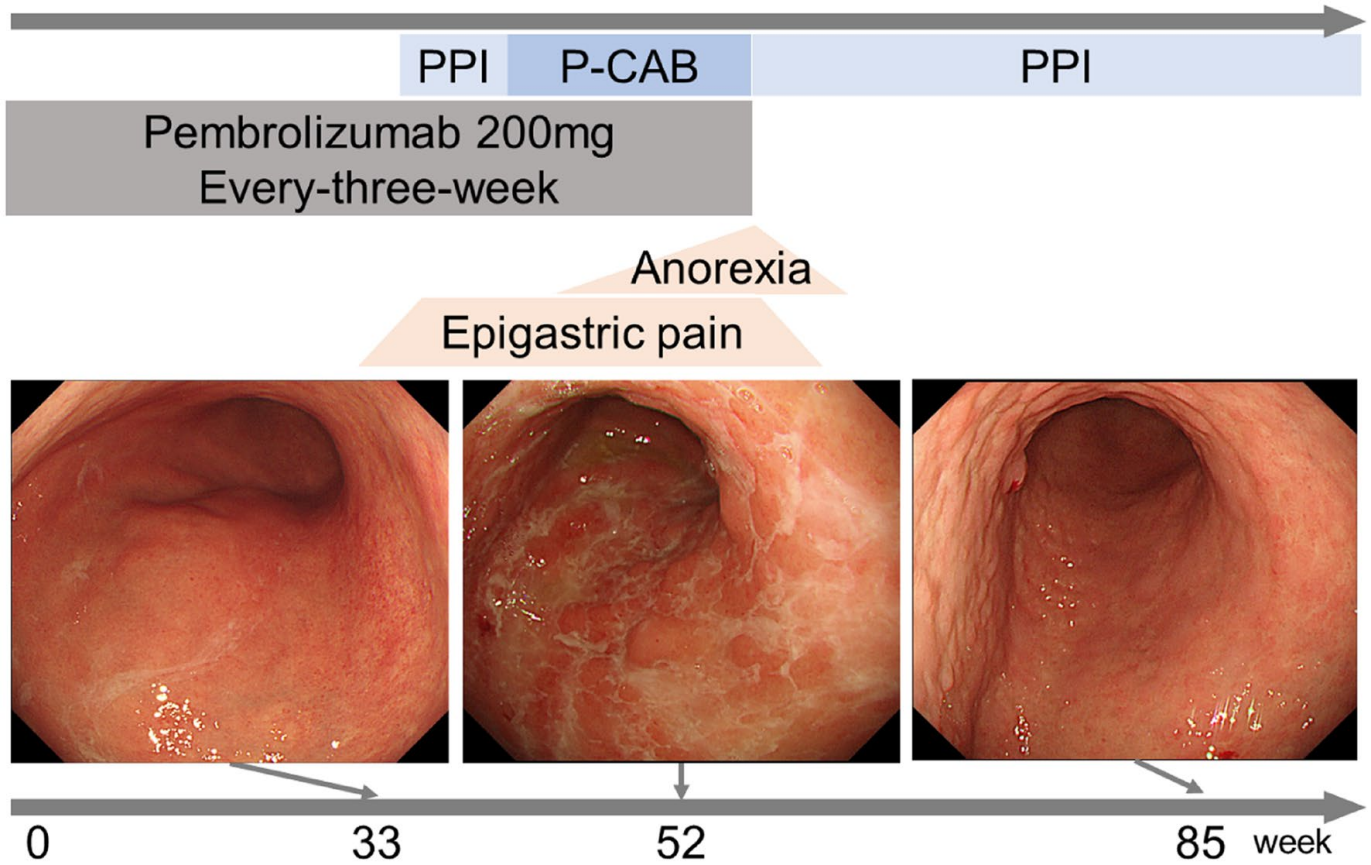
Course of clinical symptoms and endoscopic imaging findings since the administration of pembrolizumab. After the appearance of gastrointestinal symptoms, proton-pump inhibitors (PPI) and potassium-competitive acid blockers (P-CABs) were administered, but the symptoms persisted. The discontinuation of pembrolizumab at 55 weeks after the first dose resulted in symptom relief and improvement. Endoscopic examination also showed the disappearance of mucosal redness and white exudate

The patient was treated for *Helicobacter pylori* infection 3 years ago before starting pembrolizumab therapy, and the blood test results for antibodies to *H. pylori* were negative. There was no history of non-steroidal anti-inflammatory drug use or radiation therapy. Unfortunately, we did not test the patient for Cytomegalovirus (CMV) antibody titer or measured the gastrin level in the blood. Based on the clinical course, we strongly suspected ICI-related drug-induced gastritis. Therefore, pembrolizumab was discontinued, and the patient’s gastrointestinal symptoms improved within 1 week after discontinuation. Endoscopic observation of the stomach was performed 2, 5, and 9 months after discontinuation of pembrolizumab, and improvement in gastric mucosal findings was confirmed. Furthermore, by performing mucosal biopsies at each endoscopic observation, we confirmed a decrease in the number of CD8-positive T cells and an increase in the CD4/CD8 ratio in the mucosa (Fig. 3). The gastrointestinal symptoms and mucosal findings improved only with the discontinuation of ICI and administration of antacids, so steroids were not used. The patient’s gastrointestinal symptoms have not recurred more than 1 year after the discontinuation of the treatment (Fig. 4). Although the primary lung lesion is slowly worsening, the patient is being monitored by the attending respiratory physician without additional anticancer therapy.

## Discussion

In cancer treatment with ICI, irAEs are a potential cause of concern for many patients and clinicians. In terms of their frequency, gastrointestinal toxicity has been found to be the second-most common irAE after dermal toxicity [2]. However, the exact frequency of gastritis caused by ICI is unknown because there have been only 17 reports to date, including the present case [3–16].

Among these reported cases (including our report), the male-to-female ratio was 9:8, the median age was 68 years, and the median time of onset was 24 weeks (Table 1). The primary diseases were malignant melanoma in nine patients and lung cancer in seven patients. Nivolumab was used in eight patients and pembrolizumab in six patients. The condition of most patients improved with the use of steroids; however, as in the present case, some patients only recovered by discontinuing the ICI therapy. In the reports of steroid administration, most cases were treated with 1 mg/kg of prednisolone or methylprednisolone orally or intravenously. There were few specific descriptions of the duration of steroid administration, but all reports appeared to evaluate the effect of steroids within a short period and then taper off or terminate the treatment. In general, we believe that long-term administration of high-dose steroids is undesirable and should be promptly discontinued or tapered as symptoms and inflammation improved. If there is little improvement in the symptoms, the introduction of biologic agents such as infliximab should be considered. In our case, since the patient’s subjective symptoms were minimal and his general condition was good, we decided to discontinue drug administration and monitor his progress. If the symptoms did not improve, we would have considered administering steroids, such as prednisolone.

**Table 1:** Patients’ background and endoscopic findings of gastritis after ICI administration

Characteristic of the patients	*n* = 17	Symptoms	*n* = 17	Affected area	*n* = 12
Age, years [median (range)]	68 (16–93)	Epigastric pain	8	Antrum to pylorus	6
Male, *n*	9	Loss of appetite	6	Body to angle	5
Time of onset, weeks [median (range)]	24 (3–124)	Vomiting	5	Fundus	2
Primary Disease		Diarrhea	4	Endoscopic findings	*n* = 17
Malignant melanoma	9	Hematemesis	1	Erythema	9
Lung cancer	7	Tarry stool	1	Erosion	9
Hodgkin’s lymphoma	1	Abdominal enlargement	1	Edema	7
Administered ICIs^a^		Dysphagia	1	Diffuse adherence of white exudate	7
Nivolumab	8	Other coexistent irAEs^b^		Hemorrhagic gastritis	4
Pembrolizumab	6	Duodenitis	4	Atrophic, fragile mucosa	4
Ipilimumab	1	Esophagitis	3	Granular mucosa	3
PD-1 inhibitor	1	Terminal ileitis	2	Ulcer	1
PD-1 inhibitor + nivolumab	1	Cholangitis	1		
Outcome		Hepatitis	1		
Improvement without treatment	1				
Improvement only with discontinuation of ICI	2				
Improvement with steroid administration	13				
Improvement with the use of biologics	1				

^a^Immune checkpoint inhibitors

^b^Immune-related adverse events

The prognosis of gastritis caused by ICI appears to be relatively good, with improvement in clinical symptoms in all case reports.

As in the present case, epigastric pain and anorexia were frequently reported in previous cases. Although not observed in our case, there were many reports of patients with gastritis, esophagitis, duodenitis, and terminal ileitis as irAEs. A stand-by colonoscopy was also performed in the present case, but no mucosal abnormalities were found in the terminal ileum to the colon.

As in our case, endoscopic findings of previous reports showed that inflammation was mainly located in the area from the gastric antrum to the gastric body. Although reports of mucosal findings varied, erythema and erosion were the most common findings, followed by edema and diffuse white exudation. These were also observed in the present case, and the diffuse white exudate appeared flashy and could be considered an impressive finding of gastritis after ICI administration.

Pathological findings of previous cases included cryptitis, atrophy, and destruction of glands, with lymphocytic and neutrophilic infiltrates being especially common (Table 2). Despite a few reports of immuno-histochemical staining for CD4, CD8, etc., increased numbers of CD8-positive and CD4-positive T cells have been reported. In gastritis caused by ICI, Irshaid et al. reported a significant decrease in the CD4/CD8 ratio compared with that of normal tissues [17]. Consistent with the previous reports, in the present case, the number of CD8-positive T cells predominantly increased during the period of severe inflammation. We also observed a significant decrease in the number of CD8-positive T cells and an increase in the CD4/CD8 ratio as mucosal inflammation improved.

**Table 2:** Pathological findings of gastritis after ICI administration

Pathological findings	*n* = 14
Lymphocyte infiltration	9
Neutrophil infiltration	8
Cryptitis, crypt abscess	5
Atrophy and destruction of glands	4
Plasma cell infiltration	3
Apoptotic body	3
Eosinophil infiltration	2
Immunohistochemistry	*n* = 5
Increased CD8-positive T cells	4
Increased CD4-positive T cells	3

However, we had not completely ruled out the influence of infection. The subjective symptoms in our case (temporary epigastric pain and anorexia) were mild; although we suspected the involvement of ICI at that time, CMV infection should have been mentioned as a differential diagnosis because immunosuppression is often observed in patients receiving chemotherapy, especially ICIs. Therefore, CMV antigen tests and tissue immunostaining should have been performed to exclude the possibility of CMV infection at the time of treatment.

Other differential diseases include *H. pylori*-associated gastritis, radiation gastritis, drug-induced gastritis, and Zollinger–Ellison syndrome. This patient was treated for *H. pylori* infection, had no history of radiation therapy, and had no newly introduced drugs. However, serum gastrin levels had not been measured before; subsequently, the possibility of Zollinger–Ellison syndrome could not be completely ruled out. The possibility of malignant proliferative disease, such as cancer or lymphoma, was ruled out by the histological diagnosis and clinical course.

To our knowledge, this is the first report of observable improvements in both gastric mucosal and histopathological findings over time as ICI administration was discontinued. We believe that these results provide strong support for the idea that ICI administration causes immune abnormalities in patients. Although the presented patient had few subjective symptoms, the inflamed gastric mucosa had extensive erosions with diffuse white exudate deposits, mainly in the gastric body. Referring to previous reports, this finding occurred in more than 40% of patients with gastritis after ICI administration. In future, when patients receiving ICIs complain of abdominal symptoms, aggressive tissue biopsy and immuno-histochemical staining should be performed in addition to endoscopy to provide characteristic endoscopic and histological findings that are useful for diagnosis.

## References

[1] Reck M, Rodríguez-Abreu D, Robinson AG (2016). Pembrolizumab versus chemotherapy for PD-L1-positive non-small-cell lung cancer. N Engl J Med.

[2] Michot JM, Bigenwald C, Champiat S (2016). Immune-related adverse events with immune checkpoint blockade: a comprehensive review. Eur J Cancer.

[3] Onuki T, Morita E, Sakamoto N (2018). Severe upper gastrointestinal disorders in pembrolizumab-treated non-small cell lung cancer patient. Respirol Case Rep.

[4] Gonzalez RS, Salaria SN, Bohannon CD (2017). PD-1 inhibitor gastroenterocolitis: case series and appraisal of 'immunomodulatory gastroenterocolitis’. Histopathology.

[5] Boike J, Dejulio T (2017). Severe esophagitis and gastritis from nivolumab therapy. ACG Case Rep J.

[6] Vindum HH, Agnholt JS, Nielsen AWM (2020). Severe steroid refractory gastritis induced by Nivolumab: a case report. World J Gastroenterol.

[7] Nishimura Y, Yasuda M, Ocho K (2018). Severe gastritis after administration of nivolumab and ipilimumab. Case Rep Oncol.

[8] Gaffuri P, Espeli V, Fulciniti F (2019). Immune-related acute and lymphocytic gastritis in a patient with metastatic melanoma treated with pembrolizumab immunotherapy. Pathologica.

[9] Yip RHL, Lee LH, Schaeffer DF (2018). Lymphocytic gastritis induced by pembrolizumab in a patient with metastatic melanoma. Melanoma Res.

[10] Rao BB, Robertson S, Philpott J (2019). Checkpoint inhibitor-induced hemorrhagic gastritis with pembrolizumab. Am J Gastroenterol.

[11] Hayashi Y, Hosoe N, Takabayashi K (2021). Clinical, endoscopic, and pathological characteristics of immune checkpoint inhibitor-induced gastroenterocolitis. Dig Dis Sci.

[12] Kobayashi M, Yamaguchi O, Nagata K (2017). Acute hemorrhagic gastritis after nivolumab treatment. Gastrointest Endosc.

[13] Alhatem A, Patel K, Eriksen B (2019). Nivolumab-induced concomitant severe upper and lower gastrointestinal immune-related adverse effects. ACG Case Rep J.

[14] de la Mata DM, Busto-Bea V, Cerezo-Aguirre C (2021). Nivolumab-induced gastritis in a patient with metastatic melanoma. Rev Gastroenterol Mex.

[15] Cǎlugǎreanu A, Rompteaux P, Bohelay G (2019). Late onset of nivolumab-induced severe gastroduodenitis and cholangitis in a patient with stage IV melanoma. Immunotherapy.

[16] Vandepapelière J, Siplet J, Libbrecht L (2020). Auto-immune gastritis induced by pembrolizumab, an anti-PD-1, in a melanoma patient. Acta Gastroenterol Belg.

[17] Irshaid L, Robert ME, Zhang X (2021). Immune checkpoint inhibitor-induced upper gastrointestinal tract inflammation shows morphologic similarities to, but is immunologically distinct from, helicobacter pylori gastritis and celiac disease. Arch Pathol Lab Med.

